# Endoscopic myringoplasty: comparison of double layer cartilage-perichondrium graft and single fascia grafting

**DOI:** 10.1186/s40463-020-00440-7

**Published:** 2020-06-22

**Authors:** Zheng cai Lou

**Affiliations:** Department of Otorhinolaryngology, the affiliated Yiwu hospital of Wenzhou medical university (Yiwu central Hospital), 699 jiangdong road, Yiwu city, 322000 Zhejiang provice China

**Keywords:** Endoscopy, Myringoplasty, Double layer graft, Cartilage-perichondrium, Fascia graft

## Abstract

**Objective:**

To compare surgical results of endoscopic myringoplasty using double layer cartilage-perichondrium grafts versus single fascia grafts.

**Study design:**

Prospective, randomized, controlled.

**Setting:**

University-affiliated teaching hospital.

**Subjects and methods:**

In total,134 patients who underwent endoscopic myringoplasty were included in this study. Patients in group A received a double layer tragal cartilage-perichondrium graft and those in group B received a temporal muscle fascia graft. The groups were compared with respect to the pre- and postoperative air-bone gap (ABG) and the graft success rate.

**Results:**

The graft success rate was 98.5% (66/67) in the Group A and 94.0% (63/67) in the Group B at 6 months, the difference wasn’t statistically significant (*p* = 0.362). However, the graft success rate was 97.0% (65/67) in the Group A and 85.1% (57/67) in the Group B at 12 months, the difference was statistically significant (*p* = 0.034).

In addition, only one patient (1.49%) had small keratin pearls in the Group A, no patients developed cholesteatoma of middle ear in either group.

**Conclusions:**

The endoscopic double layer perichondrium-cartilage graft technique is feasible for repairing medium or larger perforations, it has a better long-term graft success rate and less operative time compared with the single layer fascia graft technique. However, long-term hearing outcomes were the same for the single and double layer closure techniques.

## Introduction

Chronic tympanic membrane (TM) perforation is a common complication of chronic otitis media (COM) and trauma in adult; persistent perforation requires surgical closure by myringoplasty. Several graft materials and graft placement methods are used in myringoplasty. Fascia, perichondrium, cartilage and fat grafts are often used, but fascia is still the preferred graft material for most patients. However, cartilage grafts are favored in difficult cases, such as those with poor Eustachian tube function, retraction pockets, infection, or anterior perforations, as well as in revision surgery [1, 2]. The graft can be placed as underlay or overlay with an endaural or postauricular approach [3]. The underlay technique is associated with a higher risk of failure of the fascia graft due to lack of adequate graft stabilization and poor blood supply, and of subsequent failure of closure for large or marginal perforations [4–6]. The lateral graft technique is effective in repairing perforations and can achieve excellent hearing outcomes, but requires extensive canalplasty and carries an added risk of TM blunting, lateralization, and excessive TM thickness [7, 8]. An endoscopic approach has become popular in aural surgery [4–6]. Endoscopy provides adequate visualization and the perforation margins can be clearly evaluated, even if there are exostoses of the external auditory canal (EAC).

Endoscopic inlay butterfly cartilage tympanoplasty, without the need for canalplasty or elevation of the tympanomeatal flap, has an excellent graft success rate, although it is technically more challenging in terms of achieving precise measurement of the perforation size and shape, and accurate sizing of the cartilage graft [9–12]. In this study, we introduced a double layer cartilage-perichondrium graft, a novel modified over-underlay technique for the reconstruction of TM. This technique combines the ease of the underlay technique with the overlay approach, taking tragal cartilage with perichondrium, freeing the edges of the perichondrium, and placing the graft through the perforation, with the cartilage medial to the perforation and the perichondrium lateral. This study compared the anatomical and functional outcomes in TM perforations of double layer cartilage-perichondrium graft and underlay fascia graft via endoscopy.

## Materials and methods

### Ethical considerations

The study protocol was reviewed and approved by the Institutional Ethical Review Board of the affiliated yiwu hospital of wenzhou medical university (Yiwu Central Hospital), Yiwu city, Zhejiang provice, China. Informed consent was obtained from all participants.

### Patients and methods

A prospective interventional randomized case series study was conducted from 2014 to 2018 in a tertiary care hospital. Inclusion criteria required patients to be older than 18 years, to have a diagnosis of unilateral TM perforation with COM, without cholesteatoma and with an intact ossicular chain, and to require TM repair. Patients with suspicious ossicular chain disruption, middle ear inflammation, myringitis and revision cases were excluded from the study.

Pure-tone audiometry (PTA) was measured preoperatively and 6 months after surgery. Standard PTA was performed for the frequencies of 0.5, 1, 2 and 3 kHz. The air-bone gap (ABG) was calculated as the average difference between air conduction and bone conduction at 0.5, 1, 2, and 3 kHz. The patient with ossicular chain disruption was suspected if the preoperative ABG was more than 40 dB, who was then excluded from the study. Age, sex, presence of contralateral perforation or otitis media with effusion (OME), size, location of perforation, and preoperative and postoperative hearing levels were recorded for all patients. The location of perforation was described as marginal in cases in which the annulus was involved regardless of involvement of the umbo of the malleus; otherwise, it was described as central, then, the marginal perforations were classified as follows in relation to the handle of the malleus: antero-marginal, postero-marginal or inferomarginal. The TM perforation was classified according to size, as subtotal (> 75% of the eardrum), large (> 50%), or medium (25–50%). The small perforation wasn’t included in this study because fat graft or perichondrium graft myringoplasty was usually used for the small perforation in our department.

Allocation of the technique and material of graft were performed by operating room nurse (ORN) using simple random sampling. Specifically, consecutive subjects who met the inclusion criteria and signed the consent form assigned random numbers generated by the SPSS for Windows software package (ver. 19.0; SPSS, Inc., Chicago, IL, USA) that allocated them to one of the two groups: double layer cartilage-perichondrium graft group (Group A) and temporal fascia graft group group (Group B). Surgery was performed by the same surgeon.

### Surgical procedure

#### Endoscopic myringoplasty

A 0° angle, 4-mm-wide and 18-cm-long rigid endoscope (Hangzhou Tonglu Cutting-edge endoscope Co. LTD, Tonglu city, China) and high-definition monitor system (XiON GmbH) were used in all cases. All patients were operated on under general anesthesia. No postauricular approaches were performed. The perforation edges were visualized and deepithelialized with an angled pick.

#### Tragal cartilage-perichondrium graft

**Fig. 1:**
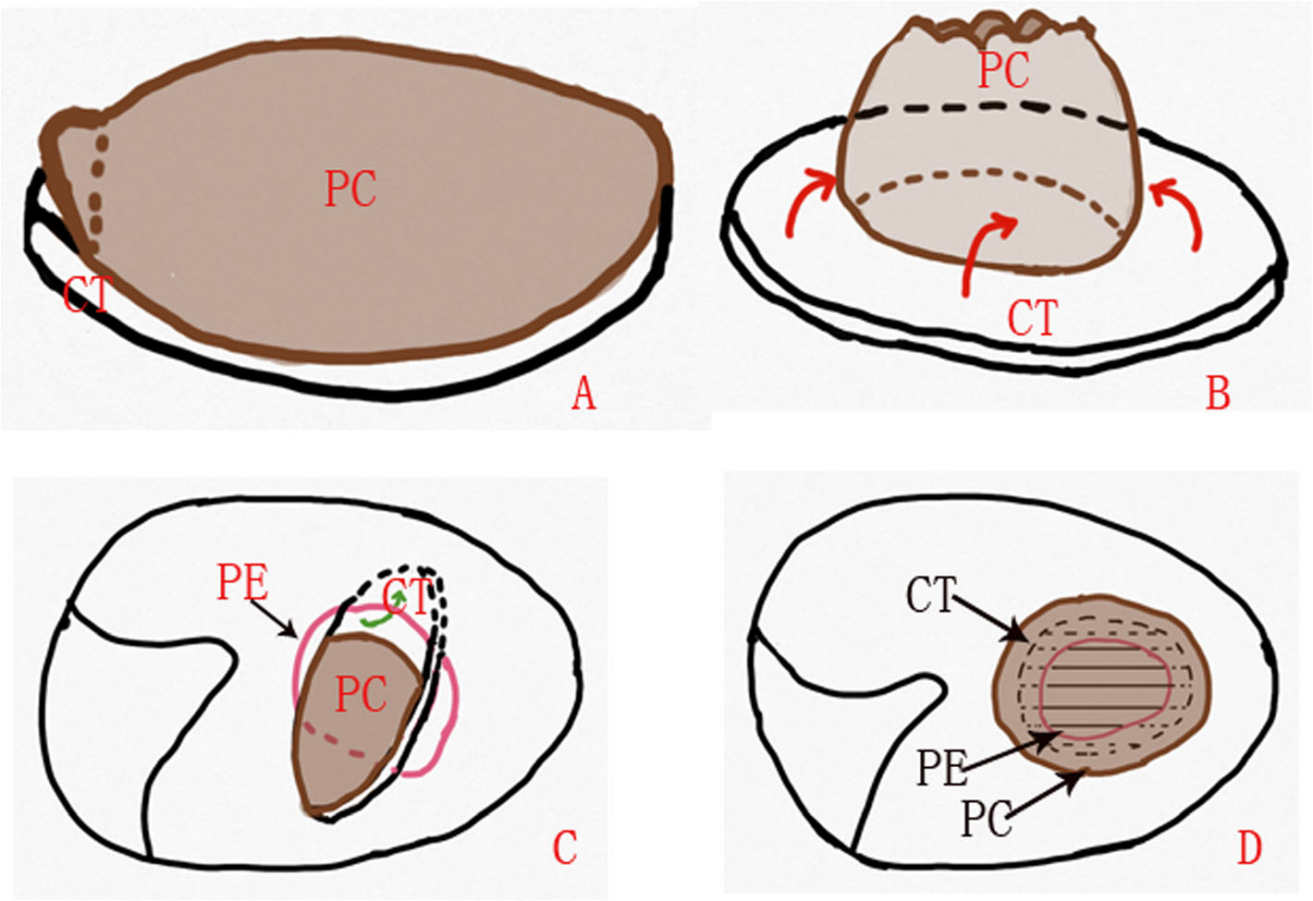
Diagram of double layer cartilage-perichondrium graft myringoplasty. The preparation of tragal cartilage with a single layer perichondrium (**a**), the stripping of lateral perichondrium (**b**), the cartilage graft was placed trans-perforation medial to the TM remnant (**c**), the lateral perichondrium was placed lateral to the TM remnant (**d**). PC, perichondrium; CT, cartilage; PE, perforation edge

**Fig. 2:**
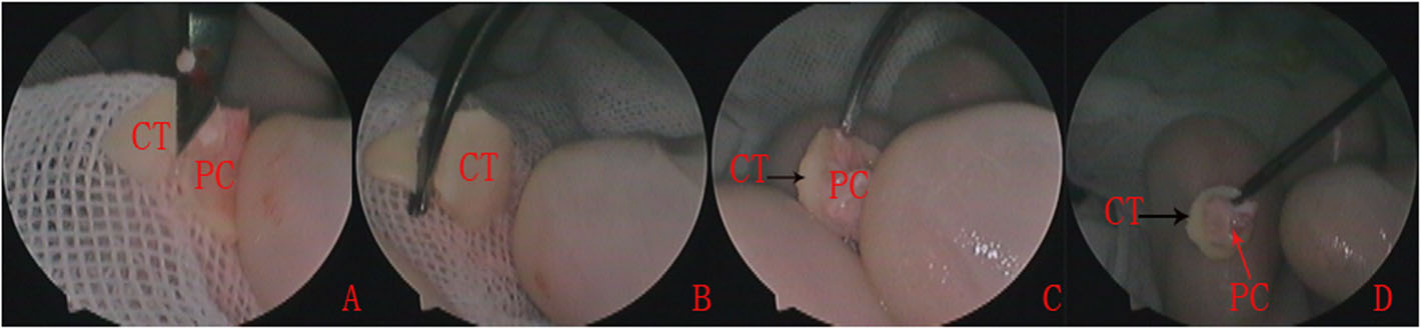
Preparation of a double layer tragal cartilage perichondrium graft. The tragal cartilage with a single layer of perichondrium was harvested (**a**), the tragal cartilage graft was shaped (**b**), and the lateral perichondrium was peeled circumferentially and rolled up from the cartilage graft (**c** and **d**). PC, perichondrium; CT, cartilage

In the double layer cartilage-perichondrium group, tragal cartilage with a single layer perichondrium was used as the graft material and a 1-cm skin incision was made on the medial side of the ipsilateral tragus. An inferior cut was made as low as possible to gain the whole tragal cartilage-perichondrium; the graft was then shaped according to the perforation (Figs. 1 and 2).

The lateral perichondrium was peeled circumferentially and rolled up from the cartilage graft, forming a double layer graft with an interconnecting pedicle. The lateral graft is the perichondrium and the medial graft is the intact cartilage. The size of the pedicle of the perichondrium attached the center of the cartilage, which was kept intact, was equal to or less than that of the perforation.

The cartilage graft was trimmed peripherally according to the size and type of perforation, but was not thinned and was at least 2 mm larger than the perforation. A notch was made in the cartilage graft to accommodate the exposed malleus handle, if applicable. The lateral free perichondrium was also trimmed peripherally, but was at least 1–2 mm larger than the medial cartilage graft (Fig. 2).

**Fig. 3:**
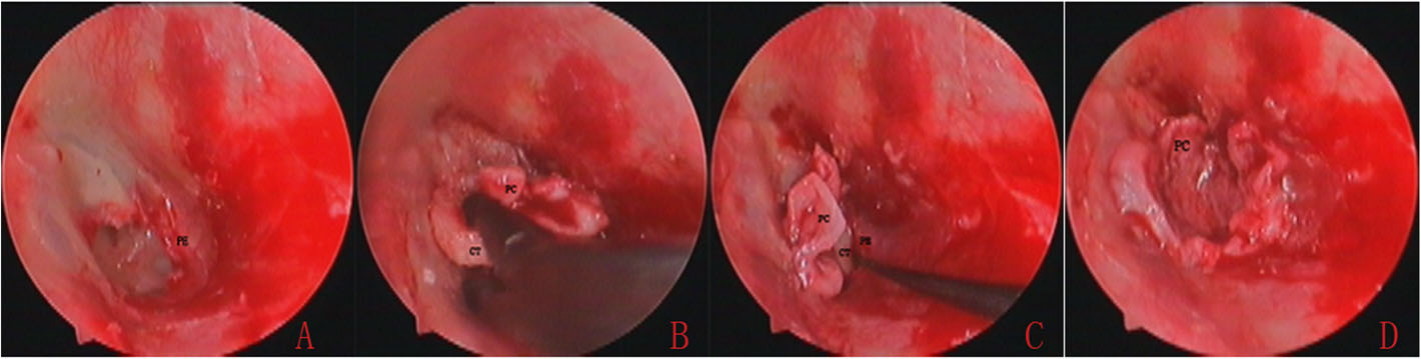
The perforation edges were deepithelialized (**a**). The cartilage-perichondrium graft accessed to the perforation via the EAC (**b**). The cartilage graft was placed medial to the remnant TM and annulus (**c**). The lateral perichondrium was elevated and placed lateral to the malleus, remnant TM, and annulus (**d**).PC, perichondrium; CT, cartilage; PE, perforation edge

The medial cartilage graft was placed trans-perforation medial to the TM remnant and the annulus; a notch of cartilage was accommodated the malleus to occlude the perforation. The lateral perichondrium was placed lateral to the malleus, annulus, TM remnant and EAC. However, the lateral surface of the TM around the perforation edges wasn’t de-epithelialized over a large area. No tympanomeatal flap elevation was performed for any perforation. Biodegradable Nasopore (biodegradable NasoPore, Stryker, Kalamazoo, MI, USA) soaked in erythromycin ointment was used to support the graft, medially and laterally. The EAC was packed with gauze soaked in erythromycin ointment up to the tragus incision, which was not sutured (Fig. 3).

#### Temporalis fascia graft

In the fascia graft group, a temporalis fascia graft of adequate size was harvested via a small incision just above the ear, and then pressed using a fascia clamp. The incision was sutured using 3–0 absorbable suture material. The fascia graft was placed medial to the TM remnant and the annulus in an underlay fashion without TM flap elevation in 31 patients with medium size central perforation. However, a tympanomeatal flap was elevated according to the localization and size of the perforation in 36 patients with marginal and large perforations. Then, the fascia graft was placed accordingly to close the perforation with the over-underlay technique or underlay fashion. The tympanomeatal flap was repositioned.

Biodegradable Nasopore soaked in erythromycin ointment was used to support the graft medially and laterally in all the patients. The EAC was packed with gauze soaked in erythromycin ointment up to the tragus.

#### Postoperative follow-up

Postoperatively, all the patients received oral amoxicillin/clavulanate potassium for 1 week to prevent infection. Postoperative follow-up visits took place at the hospital at weeks 2, 3 and 4 and at months 3, 6, 12, and 18. The packing gauze was removed from the EAC at 14 days post-surgery and biodegradable Nasopore fragments were aspirated from the EAC at 3–4 weeks post-surgery; this allowed the graft to be visualized endoscopically.

### Outcome assessment

In both groups, the operating time (from start of surgery after anesthesia induction to EAC packing) was recorded. Endoscopic examination was performed at each follow-up visit to view the graft change. The development of cholesteatoma and epithelial pearls were evaluated by each endoscopic examination and Computed tomography at 18 months postoperatively. An audiometric evaluation was carried out to measure the ABGs at the end of postoperative month 6 and 12. Any intraoperative or postoperative complications were also recorded. Graft success was defined as an intact graft without perforation, retraction, lateralization, significant blunting or medialization. The residual perforation was defined as that a perforation was seen following the removal of biodegradable Nasopore fragments. However, the re-perforation was defined as that the TM had healed completely following surgery but new perforation occured at postoperative 3 months.

### Statistical analysis

Statistical analyses were performed with SPSS software (version 21.0; SPSS Inc., Chicago, IL, USA). The data are expressed as means (standard deviation [SD]) and percentages (%). The chi-squared test was used for comparison of categorical data. Wilcoxon and Mann-Whitney U tests were used for analyzing non-parametric variables; independent and paired-samples t-tests were used for parametric variables. Results with a *p*-value < 0.05 were accepted as statistically significant.

## Results

### Patient characteristics

A total of 134 patients with 134 ears (81 females, 53 males; 134 affected ears) who met the above criteria were included in this study. The average age was 35.1 ± 1.29 years. The left ear was affected in 72 (53.7%) patients, and the right ear in 62 (46.3%) patients. Of the 134 ears, 67 were randomized to the double layer graft group (Group A) and 67 to the fascia graft group (Group B). Patient characteristics are shown in Table 1. The age, sex, side of affected ear, size, location of perforation, the preexisting myringosclerosis, status of non-operated ear, smoking status, and presence of diabetes were matched between the groups (*p* > 0.05). No ossicular chain disruption was found during surgery in all the patients in either group. Elevation of the tympanomeatal flap was performed in 36 (53.7%) patients in the B group. The average operating time was significantly shorter in the A group compared with the B group (36.4 ± 3.8 vs. 62.7 ± 4.2 min, *p* < 0.05). In addition, the mean surgical time was also significantly shorter in the cases without TM flap elevation compared with the cases with TM flap elevation in the fascia group (48.6 ± 6.2 vs. 87.3 ± 5.1 min, *p* < 0.05). All tragal incisions had healed by postoperative week 2 in the double layer graft group. All patients were followed up at least 18 months. The mean follow-up period was 20.2 ± 1.9 months in the Group A and 21.3 ± 2.6 months in the Group B (Table.1).

**Table 1:** Demographic characteristic of patients between the groups

	double layer graft group	fascia graft group	*P* value
No.	67	67	
Sex (F:M)	39:28	42:25	0.891^a^
Age (years)	34.6 ± 1.30	35.0 ± 2.07	0.537^b^
Location of perforation (Central vs Marginal)	41:26	45:23	0.672^a^
Marginal perforation (Antero-: Postero-: İnfero-)	13:9:4	11:7:5	0.838^a^
Size of perfortion (subtotal: large:Medium)	13:20:34	11:25:31	0.729^a^
Side of ear (L:R)	35:32	37:30	0.962^a^
Myringosclerosis (Y:N)	17:50	11:56	0.288^a^
Status of non-operated ear (contralateral perforation or OME:N)	3: 64	1: 66	0.611^a^
Smoking status (Y:N)	12:55	9:58	0.634^a^
Presence of diabetes (Y:N)	2:65	4:63	0.676^a^
Elevation of the tympanomeatal flap (Y:N)	0:67	36:31	0.001^a^
Average operating time (minutes)	36.4 ± 3.8	62.7 ± 4.2	0.001^b^
Mean follow-up period (months)	20.2 ± 1.9	21.3 ± 2.6	0.873^b^

^a^Chi-square test

^b^Independent Samples Test

### Hearing gain

The pre- and post-operative ABG showed in the Table.2. The mean ABG was pre- 23.26 ± 8.34 db, post- 6 months 11.35 ± 3.27 dB, and post-12 months 9.61 ± 2.54 db in the Group A; the difference was statistically significant (*p* = 0.001). In the Group B, the mean ABG was pre-24.15 ± 7.84 dB, post-6 months 10.12 ± 2.43 dB and post-12 months 9.43 ± 3.28 dB; again, the difference was statistically significant (*p* = 0.001).

**Table 2:** Comparison of hearing gains and the air-bone gap ((dB) mean ± SD)

	Pre-ABG	Post- ABG (6 months)	P^1^	Gain (mean)	Post- ABG (12 months)	P^1^	Gain (mean)
Double layer graft group (*n* = 67)	23.26 ± 8.34	11.35 ± 3.27	0.001^*^	12.12 ± 5.92	9.61 ± 2.54	0.001^*^	14.17 ± 4.66
Fascia graft group (*n* = 67)	24.15 ± 7.84	10.12 ± 2.43	0.001^*^	14.41 ± 7.58	9.43 ± 3.28	0.001^*^	15.27 ± 6.32
P^2^	0.794	0.832		0.836	0.895		0.811

^1^Paired Samples test ^2^Mann Whitney U test

*p < 0.01

^1^; Comparison ABG between the same groups pre- and postoperatively

^2^: Comparison between two groups in terms of gain, pre- or postoperatively

There was no statistically significant difference between the Group A and Group B in pre- (*p* = 0.794) or post-6 months ABG values (*p* = 0.832) or post-12 months ABG values (*p* = 0.895). Also, no significant group difference was found in the mean post-6 months ABG gain (*p* = 0.836) or post-12 months ABG gain (*p* = 0.811).

At post-operative 6 months, the difference was not significant for the hearing success rate of ABG ≤ 20 dB between Group A and Group B (94.0% vs 91.0%, *P* = 0.742), however, the hearing success rate of ABG ≤ 10 dB in the Group B was significantly higher than that in the Group A on paired-samples test (88.1% vs 70.1%, *p* = 0.019).

At post-operative 12 months, the difference of the hearing success rate was not significant between Group A and Group B regardless of ABG ≤ 20 dB (95.5% vs 86.6%, *P* = 0.232) or ≤ 10 dB (86.6% vs 83.6%, *P* = 0.808) (Table.3).

**Table 3:** Comparison of success rates between the groups

		Double layer graft group	Fascia graft group	*P* value
	No	67	67	
6 months	Graft take	98.5% (66/67)	94.0% (63/67)	0.362
	Hearing success (ABG ≤ 20 dB)	94.0% (63/67)	91.0%(61/67)	0.742
	ABG ≤ 10 dB	70.1%(47/59)	88.1%(59/67)	0.019
12 months	Graft take	97.0% (65/67)	85.1% (57/67)	0.034
	Hearing success (ABG ≤ 20 dB)	95.5% (64/67)	86.6% (58/67)	0.232
	ABG ≤ 10 dB	86.6% (58/67)	83.6% (55/67)	0.808

p, paired samples test, < 0.05 statistically significant differences between the groups

### Graft success rate and complications

The graft success rate was 98.5% (66/67) in the Group A and 94.0% (63/67) in the Group B at 6 months, the difference wasn’t statistically significant (*p* = 0.362). However, the graft success rate was 97.0% (65/67) in the Group A and 85.1% (57/67) in the Group B at 12 months, the difference was statistically significant (*p* = 0.034) (Table.3).

In the Group A, the residual pin-hole perforation was seen in one patient and the small re-perforation in the other patient. In the Group B, the residual pin-hole perforation was seen in four patients while graft medialization (*n* = 1) and re-perforation (*n* = 5) occured in the other 6 patients (Table 4).

**Table 4:** Comparison of the complications and findings during post-operative follow up between groups

	double layer graft group	fascia graft group
No.	67	67
Crust formation	67	0
Residual perforation^a^	1	4
re-perforation^b^	1	5
graft medialization	0	1
keratin pearls	1	0
Middle ear cholesteatoma	0	0

^a^A perforation was seen following the removal of biodegradable Nasopore fragments. ^b^The TM had healed completely following surgery but new perforation occured at postoperative 3 months.

**Fig. 4:**
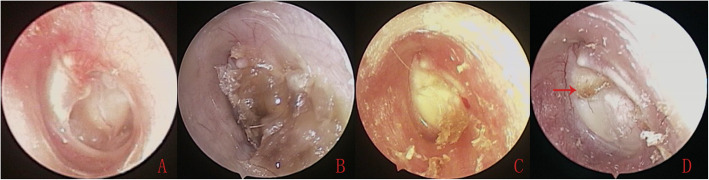
The preoperative perforation (**a**), at 3 months postoperatively (**b**), at 6 months postoperatively (**c**), epithelial pearls (Red arrow) at 19 months postoperatively (**d**)

**Fig. 5:**
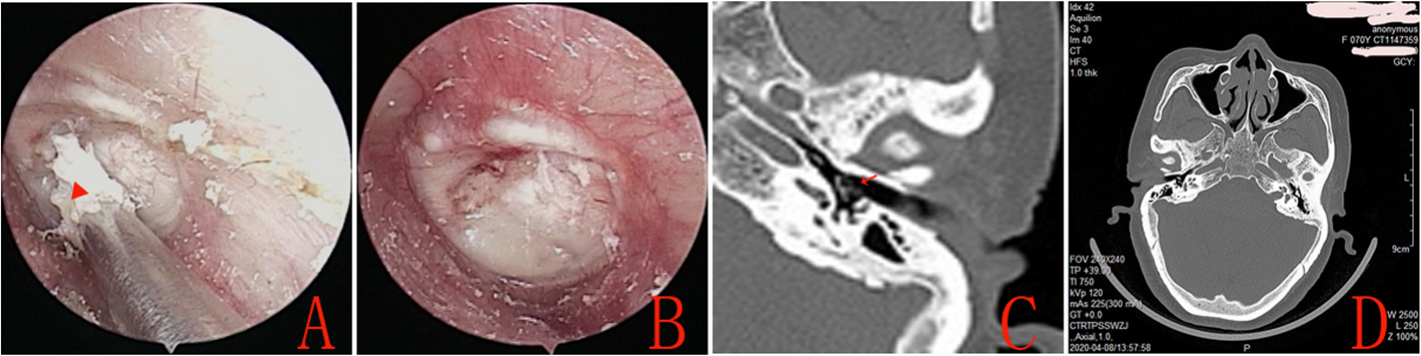
The removal of epithelial pearls (Red triangle) under endoscope (**a**), the eardrum following the removal of epithelial pearls (**b**), CT revealed the eardrum (Red arrow) and well pneumatized middle ear (**c** and **d**). Please note, this is the same patient as in Fig. 4

No other complications, such as iatrogenic sensorineural hearing loss, facial nerve palsy, vertigo, or tinnitus, graft lateralization or significant blunting were observed during the follow-up period in either group. Endoscopic examination found that the lateral perichondrium graft gradually formed the crust in all the patients in the Group A during the mean follow-up period of 20.2 ± 1.9 months (Fig. 4). However, only one patient (1.49%) had small keratin pearls on the anterosuperior quadrant in the double layer graft group, that was removed under endoscopy (Fig. 5). Nevertheless, CT revealed the well pneumatized middle ear, no patients developed cholesteatoma of middle ear in either group during the follow-up period.

## Discussion

There is no established consensus in terms of the type of surgical technique and graft material to be used for myringoplasty, or regarding postoperative care. The use of cartilage grafts has become widespread and they are frequently applied in cases of COM. Previous studies compared the anatomical and functional success rates of cartilage and fascia grafts. Özdamar et al. [13, 14] reported no significant difference in graft retention rate between their cartilage and fascia groups, as well as no significant group difference in functional success. Likewise, Bhattacharya et al. [15] reported no significant difference in the morphologic success rate between their cartilage and fascia groups. Yung et al. [16] reported no significant difference in the graft take rate between their fascia and cartilage groups (84.2% vs 80%).

In the present study, although the graft success rate wasn’t significantly different (98.5% vs 94.0%, *p* = 0.362) at 6 months between the double layer graft and fascia graft groups, the graft success rate in the Group A was significantly higer than that of the Group B (97.0% vs 85.1%, *P* = 0.034) at 12 months. These results were similar to those of previous studies. Guler et al. [17] reported that the graft success rate was 76.2% in their fascia group, but was significantly higher, at 93.5%, in the cartilage group (*p* = 0.048). Similarly, Kolethekkat et al. [18] reported a graft healing rate of 94.7% in their cartilage group, versus 70% in the fascia group. Mohanty et al. [19] reported a graft success rate of 91.95% in their cartilage group, versus 79% in the fascia group. A meta-analysis of studies comparing cartilage with fascia tympanoplasty reported overall graft integration rates of 92 and 82%, respectively (*p* < 0.001) [20]. A cartilage graft is stiff, and resistant to retraction, negative middle ear pressure and infection; it also retains its viability and shape for a long time in the presence of middle ear pathologies [21]. Although the success rate of fascia grafts is lower compared to cartilage or perichondrium grafts in most of studies [17–20], we performed a double layer cartilage-perichondrium graft myringoplasty in this study. Cartilage graft with perichondrium still attached was placed medial to the remnant TM and annulus, while the lateral free perichondrium was placed lateral to the malleus, remnant TM, annulus and even the EAC. The cartilage-perichondrium graft was still connected by a pedicle of perichondrium; this ensured that the double layer graft material completely covered the perforation and avoided positional variance. The double layer graft technique also strengthen the stability. The cartilage continuously receives nourishment through diffusion from the surface, a process facilitated by the perichondrium [22].

This technique is similar to the butterfly technique; however, rather than splitting the cartilage, the perichondrium graft is used to support the cartilage graft. On the other hand, the perichondrium graft covers the surface of remnant TM and serves as a scaffold to guide the migration of the outer squamous epithelium [23]. The procedure isn’t complicated, that may be performed by the ear surgeon with experienced endosocopic technique and microscopical middle ear surgery. Similarly, this technique would be feasible to perform with the microscope if the perforation edges are sufficiently exposed. In addition, although the small perforation wasn’t included in this study, this technique can also be applied to repair smaller perforations.

Some studies reported a high graft success rate using butterfly cartilage myringoplasty [9–12, 22]. Other studies performed the double-layered graft technique. Bedri et al. [24] reported a graft success rate of 76% with a single layer perichondrium graft, 78% with a single layer perichondrium with cartilage island graft, and 90% with a double layer (one layer with a free tragal perichondrial flap and the other with a tragal perichondrial flap to which a cartilage island remains attached). Nemade et al. [25] reported a graft take-up success rate of 97.9% using a double-layered graft with underlaid fascia and overlaid areolar fascia, versus 83.3% with an underlay fascia technique and 95.8% with an underlay cartilage technique. Thus, double layer closure was superior to single layer closure. The double-layered technique maintains intimate contact of the graft and remnant TM, and adequate stability of the cartilage graft.

Tympanomeatal flap elevation is one of the most fundamental steps during most of myringoplasty for repairing large or marginal perforations [3, 14, 26]. The tympanomeatal flap elevation may increase the fascia graft support and avoid the risk of fall for the marginal perforations [3]. However, the tympanomeatal flap elevation not only require a longer duration of surgery but also increase the bleeding of EAC and thereby affect the endoscopic operation field [11, 14]. In this study, we did not elevate the tympanomeatal flap for any perforation in the double layer graft group while the tympanomeatal flap elevation in 53.7% of cases in the fascia group. The average operating time was significantly shorter in the double layer graft group compared with the fascia graft group. The mean surgical time was also significantly shorter in the cases without TM flap elevation compared with the cases with TM flap elevation in the fascia group. In addition, some authors believed that without or limited tympanomeatal flap elevation would result in rapid wound healing and increase graft retention success with the continuation of blood perfusion [14, 26]. Tympanomeatal flap elevation was not necessary with the butterfly technique [9–12, 22]. A cartilage composite graft can easily be harvested and shaped from the existing surgical field; it does not require hair shaving or extensive dissection. Although the tragus incision was not sutured in this study, it was reached by the packing gauze in the EAC.

Almost all studies including fascia and cartilage groups reported no difference between them in terms of the functional results of the myringoplasty [13–20]. Pre- and postoperatively, no statistical difference was found in the mean ABG gain between our double layer cartilage graft and fascia graft groups. However, in present study, although the hearing success rate of ABG ≤ 20 dB was similar among two groups (94.0% vs 91.0%) at 6 months, the hearing success rate of ABG ≤ 10 dB in the fascia graft group was significantly higher than that of the double layer cartilage graft group (88.1% vs 70.1%, *P* = 0.019) at 6 months. Nevertheless, the difference of the hearing success rate was not significant between Group A and Group B regardless of ABG ≤ 20 dB (95.5% vs 86.6%, *P* = 0.232) or ≤ 10 dB (86.6% vs 83.6%, *P* = 0.808) at post-operative 12 months. This fingings were also confirmed by other study [27]. Wu PW et al. [27] suggested that large perforation may require longer time for healing and tissue remodeling between the cartilage graft and original TM, and even the malleus.

A recent meta-analysis comparing cartilage and fascia grafts [2] showed that myringoplasty using cartilage grafts had a better graft take rate than that using temporalis fascia grafts, although hearing outcomes were similar. Also, full-thickness cartilage grafts led to better hearing outcomes than temporalis fascia grafts, except in the sliced cartilage subgroup. Yegin et al. [28] found that functional results with cartilage were no different than with fascia, even though the tragal cartilage was not thinned.

One of the major concerns with lateral grafts is blunting. However, it is curious that this did not develop in this series. One possible reason is that Biodegradable Nasopore was packed for 3–4 weeks inn the EAC to prevent this from happening. Another possible reason is that the lateral perichondrium graft gradually formed the crust in all the patients in this study. We speculated that only the medial cartilage graft finally survived to close the perforation. Nevertheless, the exact mechanism is unclear, further study is expected.

The development of cholesteatoma is another concern when using the double layer cartilage-perichondrium graft technique. A theoretical disadvantage of the double layer graft technique pertains to the graft coverage of the superficial layer of the TM, which can easily hide any pathology until significant damage occurs, after which complications may manifest. The superficial layer of the TM that initially abuts the graft margins might migrate below the graft instead of covering it, which can lead to cholesteatoma. However, the lateral perichondrium graft gradually formed the crust during the mean follow-up period of 20.2 ± 1.9 months, only one patient (1.49%) had small keratin pearls on the anterosuperior quadrant, that was removed under endoscopy. Olarieta et al. [29] underwent double medial and lateral fascia graft myringoplasty and reported 4% of epithelial pearls. Fortunately, CT revealed that no patients developed cholesteatoma in either group in the current study. The present technique is similar to the butterfly technique. No cholesteatoma was observed in previous studies using the butterfly technique [9–12, 22]. Two recent double layer graft myringoplasty studies in adults also reported no cases of cholesteatoma and epithelial pearls [24, 25]. However, this study was a randomized controlled trial that used a simple randomization procedure. Also, it is sometimes difficult to distinguish the soft tissue density behind a cartilage graft using CT. Magnetic resonance imaging (MRI) could be better to monitor for long-term cholesteatoma after surgery. In addition, although the mean follow up was 18 months in this study, this follow up duration was a potential limitation, the potential risk of cholesteatoma formation will still exist in future, further follow-up should be warned.

## Conclusion

The endoscopic double layer perichondrium-cartilage graft technique is feasible for repairing medium or larger perforations, it has a better long-term graft success rate and less operative time compared with the single layer fascia graft technique. However, long-term hearing outcomes were the same for the single and double layer closure techniques.


**Additional file 1**



## Data Availability

The datasets supporting the conclusions of this article are included within the article.

## Abbreviations

COM: Chronic otitis media
EAC: External auditory canal
TM: Tympanic membrane
ABG: Air-bone gap
OME: Otitis media with effusion
CT: Computed tomography
